# TGFB1/CXCL5 axis regulation by LCN2 overexpression: a promising strategy to inhibit colorectal cancer metastasis and enhance prognosis

**DOI:** 10.3389/fimmu.2025.1548635

**Published:** 2025-04-17

**Authors:** Xiaotian Song, Shuai Xu, Dan Song, Juan Wang, Bin Bai, Yanxin An, Bin Yang, Shiqi Wang, Qingchuan Zhao, Pengfei Yu

**Affiliations:** ^1^ Department of Digestive Surgery, Xijing Hospital of Digestive Diseases, Fourth Military Medical University, Xi’an, China; ^2^ State Key Laboratory of Holistic Integrative Management of Gastrointestinal Cancers and National Clinical Research Center for Digestive Diseases, Xijing Hospital of Digestive Diseases, Fourth Military Medical University, Xi’an, China; ^3^ Department of Gastrointestinal Surgery, Key Laboratory of Hubei Province for Digestive System Disease, Renmin Hospital of Wuhan University, Wuhan, Hubei, China; ^4^ Department of General Surgery, The First Affiliated Hospital of Xi’an Medical University, Xi’an, China

**Keywords:** colorectal cancer, metastasis, LCN2, TGFB1, CXCL5

## Abstract

**Background:**

Distant metastasis remains a major reason for the high recurrence and mortality of colorectal cancer (CRC). However, the underlying molecular mechanisms driving metastasis in CRC remain poorly understood. In this study, we investigated the mechanisms underlying the inhibitory effects of lipocalin-2 (LCN2) on CRC metastasis.

**Methods:**

We assessed the expression and clinical significance of LCN2 in human CRC specimens and CRC cell lines using, immunohistochemistry, and western blot analyses. We evaluated the migratory and invasive capabilities of CRC cells influenced by LCN2 using *in vitro* transwell assays and *in vivo* lung metastatic models. RNA sequencing and proteome analysis were employed to identify potential downstream targets of LCN2. Rescue experiments were conducted to further elucidate the potential mechanisms of LCN2 and its downstream effectors in CRC.

**Results:**

LCN2 exhibited high expression levels in human CRC tissues and an inverse correlation with N classification, advanced AJCC stages, and shorter overall survival. LCN2 expression independently predicted a more favorable outcome for CRC patients. Upregulation of LCN2 effectively suppressed CRC cell metastasis both *in vitro* and *in vivo*. Mechanistically, Transforming growth factor beta 1 (TGFB1) and C-X-C motif chemokine ligand 5 (CXCL5) were identified as downstream effectors of LCN2, with LCN2 inhibiting CRC metastasis through repression of the TGFB1/CXCL5 axis. Furthermore, either TGF-βR1 inhibitor SB431542 or CXCR2 antagonist SB225002 treatment moderately decreased the migratory and invasive capabilities of DLD-1-LV-shLCN2 cells, whereas the combination treatment of the two agents dramatically decreased the migratory and invasive capabilities of DLD-1-LV-shLCN2 cells.

**Conclusions:**

This study underscores LCN2 as an independent protective factor and prognostic biomarker for CRC patients. Combined treatment with the SB431542 and the SB225002 significantly attenuated LCN2-related CRC metastasis. Targeting the LCN2/TGFB1/CXCL5 axis emerges as a promising therapeutic strategy for managing LCN2-related metastatic CRC.

## 1 Introduction

With the third highest incidence and the second highest mortality rates of all malignant tumors, colorectal cancer (CRC) is by far one of the most prevalent and most deadly (1). Globally, there were 2 million new cases and 0.9 million deaths associated with CRC in 2020, and this number is expected to rise to 3.2 million and 1.6 million by 2040 (2). For early-stage disease, surgical resection and chemotherapy are capable of curing more than 90% of CRC cases, however, once distant metastasis occurs, it is very challenging to achieve curative treatment (3). The process of metastasis involves a complex series of steps including that tumor cells escape from their primary site and establish colonies in distant tissues (4). Although significant advances have been made in biomarker-driven targeted therapies, the treatment of metastasis still lags far behind, especially for metastatic CRC (5). Thus, unveiling the molecular mechanisms underlying metastasis in CRC and identifying biomarkers are essential to develop novel targeted therapeutic approaches.

Relying on the China Gastrointestinal Proteomics Project, our research group provided hitherto undocumented evidence that pseudouridine synthase 7 (PUS7) facilitates metastasis of CRC cells by regulating LIM and SH3 protein 1 (LASP1) expression in a previous study (6). To further extend the regulatory network of PUS7 in metastatic CRC, we conducted studies on Lipocalin-2 (LCN2), whose expression was the most significantly negatively modulated by PUS7.

Lipocalin-2, also known as siderocalin, oncogene 24p3 or neutrophil gelatinase-associated lipocalin (NGAL), is a 25KDa secreted glycoprotein, acting as a carrier that primarily transports lipophilic molecules (7). LCN2 was initially characterized as a bacteriostatic factor and exerts biological function by inhibiting the siderophore-mediated iron acquiring of bacteria, thus suppressing bacterial growth (8).

An increasing body of evidence suggests that LCN2 is intricately involved in multiple physiological and pathophysiological functions, including neurodegenerative diseases (9–11), inflammatory bowel disease (12), kidney diseases (13, 14), and multiple cancers (15, 16). Most studies revealed that LCN2 is responsible for facilitating tumor progression and metastasis (15, 16). For instance, in inflammatory breast cancer, augmented levels of LCN2 reportedly promote tumor aggressiveness by regulating cell cycle-associated proteins and are correlated with a poor prognosis (15). In gastric cancer, low levels of LCN2 suppress cell proliferation, migration, invasion and cell cycle by targeting SLPI (16). Interestingly, some studies demonstrated that LCN2 contributes to inhibiting tumor development (17, 18). For instance, in oral squamous cell carcinoma, downregulation of LCN2 leads to increased survival, proliferation, migration, and chemoresistance via the LKB1-AMPK-p53-Redd1-mTOR axis (17). The discrepancies among these studies demonstrate that LCN2 represents an important driver of tumor malignant progression, but there are still some limitations. Thus, further research is warranted to elucidate the critical molecular mechanisms of LCN2.

Transforming growth factor beta 1 (TGFB1) belongs to the TGF-β subfamily and the TGF-β signaling pathway, which regulates cell fate and plays pleiotropic roles in tumor malignant progression by regulating cell proliferation, apoptosis, migration, and tumor microenvironment (TME) (19, 20). TGF-β executes its functions through two signaling mechanisms: canonical TGF-β/Smad pathway and non-canonical non-Smad pathways (including p38, phosphoinositide 3-kinase (PI3K) and mitogen-activated protein kinases (MAPKs) signaling cascades) (21). In the Smad-dependent pathway, TGFB1 triggers signaling via binding to heterotetrameric receptor complexes, composed of dimers of TGF-β type I receptors (TGFBR1) and type II receptors (TGFBR2), and subsequently phosphorylate and activate Smad2/3, which form transcriptional complexes with Smad4 and then translocate to the nucleus, regulating multiple downstream target genes (22). Substantial studies have demonstrated that TGF-β signaling pathway has a dual role in cancer, switching between tumor-suppressive and tumor-promoting phenotypes (20, 23). However, the molecular mechanisms that underlie these inconsistencies have not yet been clearly elucidated. C-X-C motif chemokine ligand 5 (CXCL5) is an inflammatory mediator that exerts a potent chemotactic effect on neutrophils by binding to the G protein-coupled receptor CXCR2 (24, 25). Previous studies have shown that the CXCL5 expression is elevated in multiple types of cancer, including colorectal cancer, breast cancer, gastric cancer, bladder cancer, and hepatocellular carcinoma (24). In colorectal cancer, CXCL5 mediates epithelial-mesenchymal transition (EMT) by activating the ERK/Elk-1/Snail and AKT/GSK3β/β-catenin signaling pathway, which promotes tumor cell migration and invasion and liver metastases (26). Besides, in breast cancer, the CXCL5/CXCR2 axis promotes tumor cell proliferation and colonization in bone (27). Taken together, these findings suggest that CXCL5 may play a pivotal and complex role in tumor progression. Nevertheless, the mechanisms of its overexpression in malignant tumors have rarely been reported, requiring more intensive investigation. Herein, we found that LCN2 was upregulated in CRC tissues and positively correlated with patient prognosis. To our knowledge, this is the first study to report that LCN2 suppresses the metastatic ability of CRC cells by inhibiting the TGFB1/CXCL5 axis. Our study also demonstrated that combination treatment with TGFB1 inhibitor SB431542 and CXCL5 inhibitor SB225002 significantly suppressed LCN2-mediated CRC metastasis.

## 2 Materials and methods

### 2.1 Public datasets

The public datasets collected in this study are publicly available from Gene Expression Omnibus (GEO) database and Gene Expression Profiling Interactive Analysis (GEPIA, http://gepia.cancer-pku.cn/). GEO and GEPIA were employed to analyze the mRNA expression level of LCN2 in human CRC specimens compared with the non-malignant specimens.

### 2.2 Cell culture

All human CRC cells (SW480, SW620, DLD-1, HCT-8, HCT-116, and RKO) were restored in our laboratory, and each cell line was tested and authenticated by short tandem repeats (STRs) DNA profiling. The above cell lines were incubated in an incubator with 5% CO_2_ atmosphere at 37°C. DLD-1, HCT-8, HCT-116, and RKO cell lines were cultured in RPMI1640 medium (Gibco, USA), while SW480 and SW620 cell lines were cultured in DMEM medium (Gibco, USA). The medium was supplemented with 10% fetal bovine serum (FBS, Gibco, USA) and 100ug/ml penicillin-streptomycin (Gibco, USA).

### 2.3 Human CRC tissue specimens and tissue microarray

12 paired fresh CRC and healthy adjacent tissues were collected from patients who underwent surgery for CRC at the Xijing Hospital of Digestive Diseases, Xi’an, China. Written informed assent was obtained from all patients involved, and ethical approval for the use of human specimens was obtained from the Xijing Hospital’s Protection of Human Subjects Committee.

HColA180Su19, a commercial human CRC tissue microarray (TMA) containing tissue specimens from 94 CRC patients (including 86 paired CRC tissues and adjacent normal tissues and 8 unpaired CRC tissues) and the associated clinicopathological information was purchased from Outdo Biotech (Shanghai Outdo Biotech, China).

### 2.4 Protein extractions and western blot

Human CRC tissues and cells were lysed in radioimmunoprecipitation assay lysis buffer (Proandy, China) containing protease inhibitor (Beyotime, China) and phosphatase inhibitor (Beyotime, China) mixture on ice to extract total proteins. Protein concentration was detected by the BCA Protein Assay Kit (Thermo Fisher, USA). The proteins were separated by SDS-PAGE (Proandy, China) and then transferred to nitrocellulose (NC) membranes (Merck Millipore, Germany). Nonspecific binding sites on the NC membranes were blocked for 1h at room temperature using 5% skimmed milk in TBS-Tween 20 (TBST, Proandy, China). Afterward, the blots were incubated overnight at 4°C with the appropriate concentration of the specific primary antibody. The following day, the membranes were rinsed three times with TBST and then incubated with the corresponding horseradish peroxidase (HRP)-conjugated secondary antibodies (Zhongshan Golden Bridge Biotech, China) for 1h at room temperature. UltraSignal enhanced chemiluminescent (ECL) reagent kit (4A Biotech, China) was then used to visualize the protein bands in a Bio-Rad ChemiDoc XRS+ Imaging System. Protein quantification was performed by densitometric analysis by Image Lab software 4.0.1.

The primary antibodies were employed as follows: anti-LCN2 (1:4000, #ab125075, Abcam, USA), anti-TGFB1 (1:1000, #ab215715, Abcam, USA), anti-CXCL5 (1:5000, #ab126763, Abcam, USA), anti-GAPDH (1:20000, #10494-1-AP, Proteintech, China), anti-tubulin (1:10000, #11224-1-AP, Proteintech, China).

### 2.5 RNA extraction and reverse transcription-quantitative polymerase chain reaction

According to the manufacturer’s protocol, total RNA was extracted using the MiniBEST Universal RNA Extraction Kit (TaKaRa, #9767, Japan), and then reverse transcription was performed using the PrimeScript RT Master Mix (TaKaRa, #RR036A, Japan) to synthesize complementary DNA (cDNA). The target sequences were amplified with RT-qPCR using SYBR Premix Ex Taq II (TaKaRa, #RR820A, Japan) on the CFX96 Real-Time PCR detection system (Bio-Rad, CA, USA). Relative mRNA expression levels were normalized to the β-actin mRNA levels and were determined using the 2^-ΔΔCt^ method.

The primer sequences used in this study are listed in **Table 1**.

**Table 1:** Primer sequences used in this study.

Primer name	Primer sequences (5’-3’)
LCN2-F	CTGAGTGCACAGGTGCCG
LCN2-R	TTAGCAGACAAGGTGGGGCT
TGFB1-F	ACGTGGAGCTGTACCAGAAAT
TGFB1-R	TGAACCCGTTGATGTCCACT
CXCL5-F	ACAGACCACGCAAGGAGTTC
CXCL5-R	TCCTTGTTTCCACCGTCCAA
β-actin -F	CTCCATCCTGGCCTCGCTGT
β-actin -R	GCTGTCACCTTCACCGTTCC

### 2.6 Immunohistochemistry and evaluation

Immunohistochemistry was employed to assess the expression of LCN2 using a Tissue Microarray (TMA) slide (HColA180Su19, Shanghai Outdo Biotech, China). The following steps were undertaken: The TMA slide was placed in an incubator and heated to 65°C for 1 hour. The slide was dewaxed in xylene and subsequently rehydrated through a graded ethanol immersion process. Antigen retrieval was performed by immersing the slide in boiling citrate buffer (0.01 M, pH 6.0) for 2 minutes. To block endogenous peroxidase activity, the slide was treated with a 3% hydrogen peroxide (H_2_O_2_) solution for 10 minutes. The section was then blocked with 5% goat serum at room temperature for 30 minutes. Next, it was incubated overnight at 4°C in a moist chamber with the primary antibody against LCN2 (diluted at 1:250, #ab125075, Abcam, USA). On the following morning, the section was subjected to a 30-minute incubation at room temperature with the corresponding secondary antibodies conjugated with HRP (Zhongshan Golden Bridge Biotech, China). Immunostaining was visualized by incubating with diaminobenzidine (DAB, Zhongshan Golden Bridge Biotech, China). The section was counterstained with hematoxylin, followed by dehydration and cover-slipping. Digital images were captured using a light microscope (Olympus, Japan) equipped with a DP70 digital camera.

The IHC results of the TMA were independently scored for staining intensity by two pathologists, both of whom were blinded to patient and clinical information. Staining intensity was assessed using the following histological scoring standards: the percentage of positive cells was scored as follows: 0 (negative), 1 (1–25%), 2 (26–50%), 3 (51–75%), or 4 (76–100%). Immunostaining intensity was evaluated as: 0 (negative), 1 (weak), 2 (medium), or 3 (strong). The final scores, ranging from 0 to 12, were calculated by multiplying the intensity and extent scores. Scores between 0 and 3 were categorized as indicating low expression levels, while scores between 4 and 12 were categorized as indicating high expression levels.

### 2.7 Construction of lentivirus and stable cell lines

To overexpress *LCN2*, a fragment of cDNA was cloned into the GV358-Puro lentivirus vector, and the short hairpin RNA (shRNA) sequence was incorporated into the GV248-Puro lentivirus vector to silence *LCN2*. Corresponding negative controls (LV-NC and LV-shNC) were constructed at the same time. All lentiviral vectors were constructed and purchased by Shanghai Genechem (Genechem, China). The *LCN2* knockdown shRNA sequences was: *LCN2* shRNA: cgGGGAATGCAATTCTCAGAG. Following the instructions of the Genechem Recombinant Lentivirus Operation Manual, Hitrans P (GeneChem, China) was applied for cell transfection, which was conducted at 60% confluence with a final lentivirus multiplicity of infection (MOI) of 30-50. 72 h after infection, CRC cells were screened for using 2-5 μg/mL puromycin (OriGene, USA). The transduction efficiency of the lentivirus was confirmed using the RT-qPCR and Western blot assays.

### 2.8 *In vitro* migration and invasion assays

The migratory and invasive capacities of transfected cells were measured in 24-well transwell chambers with an 8-μm pore polycarbonate membrane filter (Corning, USA). For the migration assays, 7 × 10^4^ cells were seeded in the upper chamber after being suspended with the corresponding medium without FBS, and 600 μL of corresponding medium containing 20% FBS was added into the bottom chamber. For the invasion assays, each chamber insert was coated with 200 mg/mL of Matrigel (Corning, USA). Then, 5 × 10^4^ cells were seeded in the upper chamber after being suspended with the corresponding medium without FBS, and 600 μL of the corresponding medium containing 20% FBS was added into the bottom chamber. After incubation at 37°C for a fixed duration, the cells that had migrated or invaded to the bottom surface of the filter were fixed in 4% paraformaldehyde (Hete Biotech, China) and then stained with 1% crystal violet (Beyotime, China) for 10min. Finally, we visualized the cells that migrated or invaded the bottom surface using a microscope (Olympus, Japan) for counting the cells and statistical analysis. All assays were performed in triplicate, and the relative migratory or invasive capacities were expressed relative to the indicated control cells.

### 2.9 *In vivo* lung metastatic model

Five-week-old female BALB/c nude mice were purchased from Weitonglihua Corporation (Beijing, China) and were raised and cared for in a specific-pathogen-free environment. Animal experiments were authorized by the Ethical Committee of the Air Force Medical University. Twenty nude mice were randomly divided into the HCT-8-LV-NC group and HCT-8-LV-LCN2 group (n = 10 in each group), and sixteen nude mice were randomly divided into the DLD-1-LV-shNC group and DLD-1-LV-shLCN2 group (n = 8 in each group). *In vivo* lung metastasis models were constructed via tail vein injections. We injected 2 × 10^6^ cell suspensions in 150 μL phosphate-buffered saline (PBS, Gibco, USA) into the tail vein of mice in HCT-8 groups and 3 × 10^6^ cell suspensions in 150 μL (PBS, Gibco, USA) into the tail vein of mice in the DLD-1 groups, respectively. Eight weeks after the initial injections, the mice were sacrificed, and lung metastatic nodules were carefully counted in paraffin-embedded sections stained with hematoxylin-eosin (HE).

### 2.10 Agents

Recombinant human TGFB1 protein was purchased from MCE (#HY-P7118, China). Recombinant human CXCL5 protein was purchased from MCE (#HY-P70572, China). The TGF-βR1 inhibitor SB431542 was purchased from Selleck Chemicals (#S1067, USA). The CXCR2 antagonist SB225002 was purchased from MCE (#HY-16711, USA).

### 2.11 Quantification and statistical analysis

All statistical analyses were performed using SPSS software (version 23.0, IBM SPSS) and Prism software (version 8.2.1, GraphPad Software). Comparisons of quantitative data were compared between groups using the Student’s t-test or Mann-Whitney U test. Comparisons of categorical data were analyzed using Fisher’s exact test. Overall survival curves were estimated using the Kaplan-Meier method, and the differences between the LCN2^low^ and LCN2^high^ groups were evaluated by the log-rank test. Univariate and multivariate analyses were conducted by the Cox proportional hazards model to identify the independent affected factors for overall survival. A *p*-value < 0.05 was statistically significant.

## 3 Results

### 3.1 LCN2 is upregulated in human CRC tissues and correlates with longer overall survival

**Figure 1:**
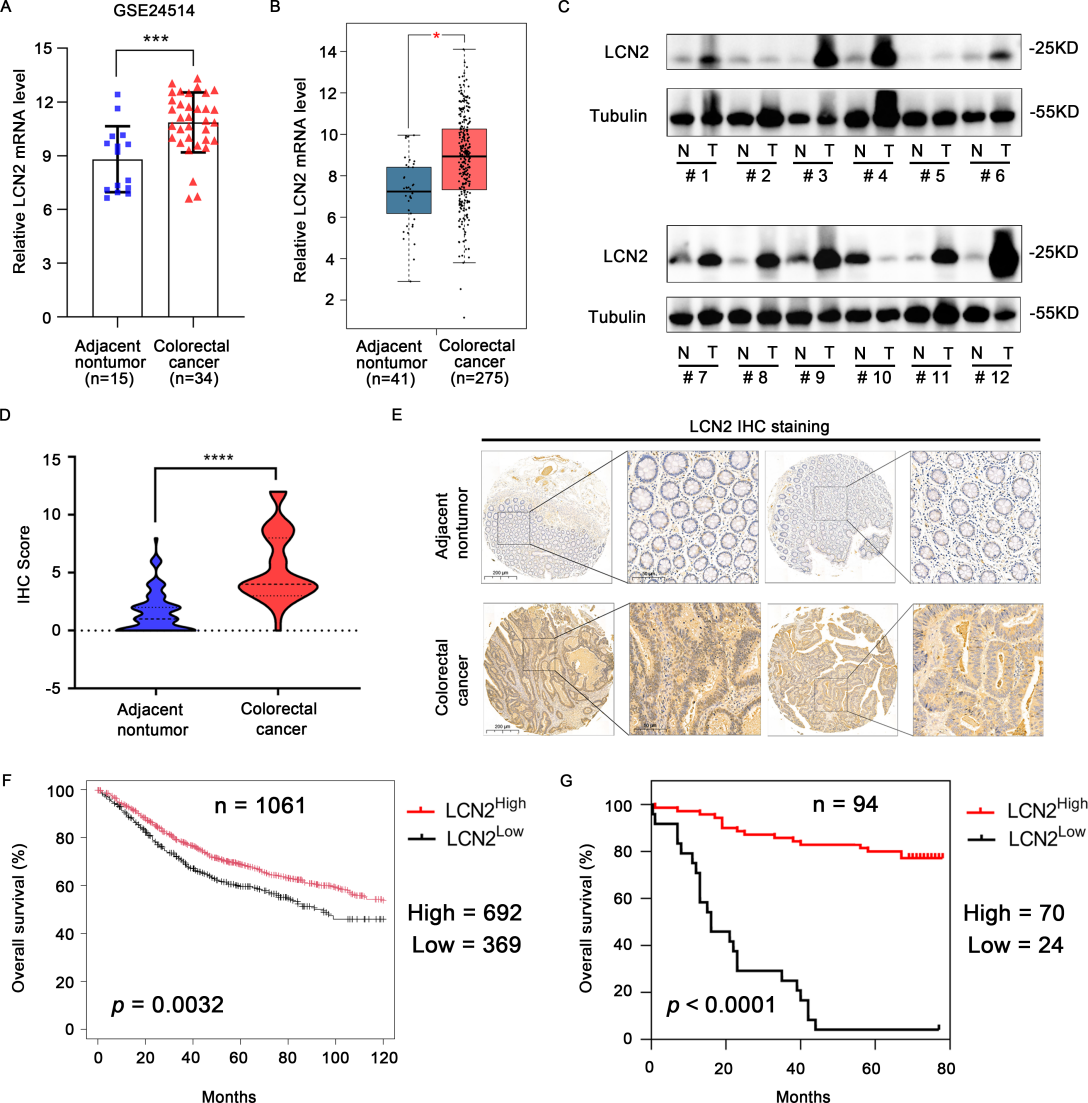
LCN2 is upregulated in human CRC tissues and predicts longer overall survival. **(A)** The transcriptional levels of LCN2 between the adjacent nontumor and CRC tissues were identified from the GEO database (GSE24514). **(B)** The transcriptional levels of LCN2 between the adjacent nontumor and CRC tissues were identified from the GEPIA database. **(C)** The protein levels of LCN2 in 12 paired adjacent nontumor and CRC specimens. **(D, E)** Plot of IHC scores of LCN2 expression in paired adjacent nontumor (n = 86) and CRC (n = 94) specimens and representative positive IHC staining. **(F)** Kaplan-Meier Plotter database analysis showed that CRC patients with high expression of LCN2 had longer OS **(G)** Kaplan-Meier survival analysis exhibited the relationship between OS and LCN2 expression in patients with CRC. Data are presented as the mean ± s.d. *p < 0.05, ***p < 0.001, ****p < 0.0001.

To investigate the expression of LCN2 in human CRC, we analyzed the mRNA expression levels in the GEO and GEPIA (28) databases. The results showed that the mRNA expression levels of LCN2 were significantly upregulated in CRC tissues compared with the adjacent normal tissues (**Figures 1A, B**). To confirm the increased expression of LCN2, the protein expression levels of LCN2 examined by western blot (WB) assay in 12 paired CRC and adjacent normal specimens revealed that LCN2 was upregulated in most patients (n = 9/12) (**Figure 1C**). To further evaluate the potential clinical value of LCN2, we performed IHC staining on a cohort of tissue microarrays, including 86 paired CRC and adjacent normal specimens and 8 unpaired CRC specimens. In this cohort, the expression of LCN2 was upregulated in approximately 74.5% (70/94) of the paired specimens (**Figures 1D, E**). Next, the relationship between clinicopathological features of CRC patients and LCN2 expression was analyzed. Correlation analysis revealed that the elevated LCN2 expression was negatively correlated with advanced AJCC stages (*p* = 0.014) and N stage (*p* = 0.017) (**Table 2**). Multivariate analysis indicated that high expression of LCN2 was an independent protective factor for CRC patients (**Table 3**). Moreover, Kaplan-Meier Plotter database analysis showed that CRC patients with high expression of LCN2 had longer overall survival (OS) (**Figure 1F**). Consistent with the database results, Kaplan-Meier survival analysis demonstrated that CRC patients from Tissue Microarray with high expression of LCN2 had a longer OS compared to those with low expression of LCN2 (*p* < 0.0001) (**Figure 1G**). Collectively, these results demonstrated that LCN2 was significantly upregulated in CRC and positively correlated with the prognosis.

**Table 2:** Correlation between LCN2 expression and clinicopathological characteristics of human CRC tissues.

Clinicopathological variables	Cases (No.) n=94	LCN2 (No.)		p-Value
		Low Expression	High Expression	
Age(y)				0.939
<60	28	7	21	
≥60	66	17	49	
Gender				0.409
Male	48	14	34	
Female	46	10	36	
AJCC stage				0.014
I	10	0	10	
II	48	9	39	
III	31	12	19	
IV	5	3	2	
T classification				0. 098
1	1	0	1	
2	10	0	10	
3	51	12	39	
4	32	12	20	
N classification				0.017
0	60	10	50	
1	25	9	16	
2	9	4	3	
M classification				0.069
0	89	21	68	
1	5	3	2	

**Table 3:** Univariate and multivariate analysis of factors associated with overall survival of human CRC.

Clinicopathological variables	Overall survival		
	p-Value	Hazard ratio	95% Confidence interval
Univariate analysis			
Age (<60 vs. ≥60)	0.903	0.959	0.486-1.893
Gender (Male vs. Female)	0.704	0.885	0.471-1.661
AJCC stage (I vs. II vs. III vs. IV)	<0.001	2.955	1.898-4.599
T classification (T1 vs. T2 vs. T3 vs. T4)	0.001	2.450	1.418-4.231
N classification (N1 vs. N2 vs. N3)	<0.001	2.582	1.718-3.883
M classification (M0 vs. M1)	0.002	4.700	1.761-12.546
LCN2 expression (Low vs. High)	<0.001	0.092	0.046-0.184
Multivariate analysis			
AJCC stage (I+II vs. III+IV)	0.363	1.656	0.559-4.910
T classification (I vs. II vs. III vs. IV)	0.225	1.481	0.785-2.794
N classification (I vs. II vs. III)	0.788	1.107	0.529-2.313
M classification (M0 vs. M1)	0.831	0.833	0.156-4.442
LCN2 expression (Low vs. High)	<0.001	0.142	0.068-0.301

### 3.2 Overexpression of LCN2 suppresses CRC metastasis *in vitro* and *in vivo*

**Figure 2:**
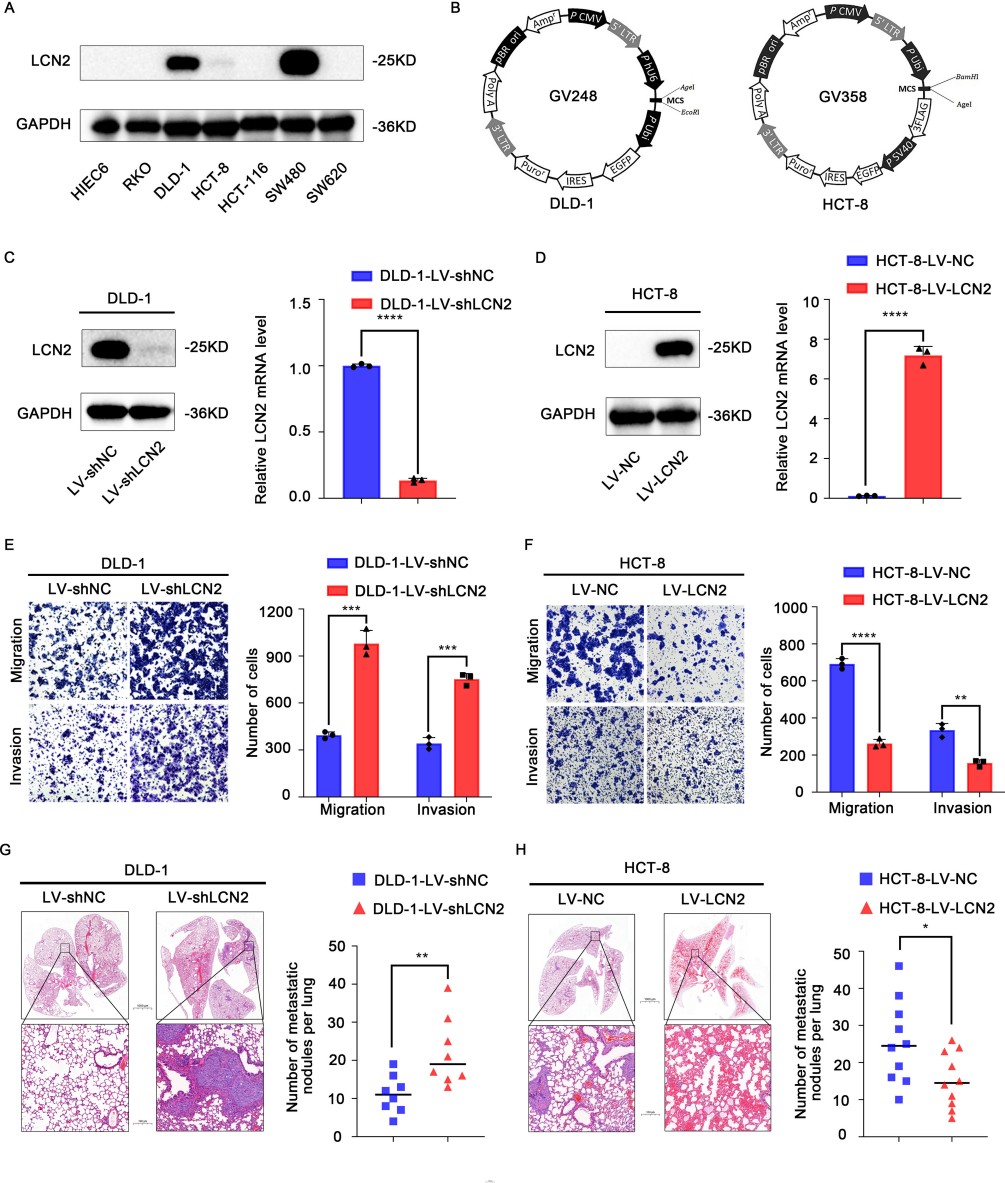
Overexpression of LCN2 suppresses CRC metastasis *in vitro* and *in vivo*. **(A)** The protein expression levels of LCN2 in HIEC6 and CRC cell lines RKO, DLD-1, HCT-8, HCT-116, SW480, and SW620. **(B)** Structure of lentiviral vectors. **(C, D)** The lentivirus transfection efficiency in DLD-1 and HCT-8 CRC cells was verified by RT-qPCR and WB. **(E, F)** Migratory and invasive capabilities assessed by transwell assay for the indicated CRC cell lines. **(G, H)** Hematoxylin-eosin (H&E) staining of lung tissues from representative mice and the number of lung metastatic foci in each group at 8 weeks. (n = 10 mice per group for DLD-1 cells and n = 8 mice per group for HCT-8 cells). Data are presented as the mean ± s.d. *p < 0.05, **p < 0.01, ***p < 0.001, ****p < 0.0001.

To gain further insights into the function of LCN2 in CRC metastasis, we examined LCN2 expression levels in HIEC6 and several CRC cell lines by western blot assay. The results indicated that LCN2 protein expression levels were upregulated in DLD-1 and SW480 cell lines and downregulated in HIEC6, HCT-8, SW620, RKO, and HCT-116 cell lines (**Figure 2A**). To unveil the role of LCN2 in CRC metastasis, HCT-8 cells with relatively low LCN2 expression and DLD-1 cells with relatively high LCN2 expression were selected to construct HCT-8-LV-LCN2 and DLD-1- LV-shLCN2 stable cell lines using recombinant lentivirus transfection (**Figure 2B**). Then, the transfection efficiency was evaluated by RT-qPCR and WB (**Figures 2C, D**).

Next, to investigate the effects of CRC cells with high and low expression of LCN2 on migratory and invasive capacities, transwell assays were performed. The results indicated that the ectopic expression of LCN2 significantly diminished the migratory and invasive capacities of HCT-8 cells, while downregulated expression of LCN2 yielded the opposite effects on DLD-1 cells, compared with the control group (**Figures 2E, F**).

Besides, to assess the effect of changes in LCN2 expression on the metastatic potential of DLD-1 and HCT-8 cells, lung metastasis models were constructed by tail vein injection in 5-week-old female BALB/c nude mice. Similar results were obtained to those observed *in vitro*; compared with the control group, LCN2 overexpression significantly decreased the number of metastatic lung nodules and lung metastasis incidence, while downregulation of LCN2 remarkably increased the number of metastatic lung nodules and lung metastasis incidence (**Figures 2G, H**). Collectively, these findings demonstrated that LCN2 functioned as a metastasis-suppressor in the migration and invasion of CRC cells.

### 3.3 Pro-metastatic proteins TGFB1 and CXCL5 are downstream targets of LCN2

**Figure 3:**
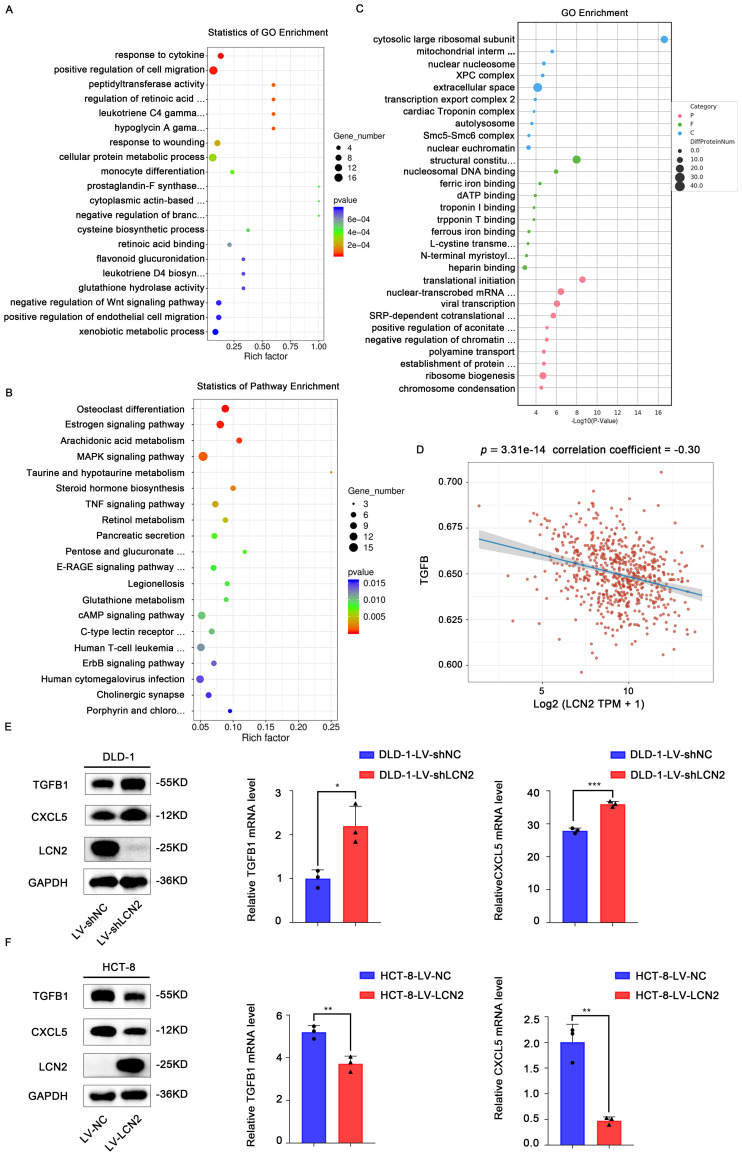
Pro-metastatic proteins TGFB1 and CXCL5 are downstream targets of LCN2. **(A, B)** Bubble plot for visualizing GO and KEGG term enrichment based on RNA-seq results. **(C)** Bubble plot for visualizing KEGG term enrichment based on proteome profiling results. **(D)** This is a Spearman correlation analysis plot, which is used to show the correlation between the TGF-β pathway score and the expression of LCN2. In this plot, the X-axis represents the distribution of the expression of LCN2, and the Y-axis represents the distribution of the TGF-β pathway score. The values at the top represent the results of the Spearman correlation analysis, including the p-value, correlation coefficient. **(E)** WB and RT-qPCR analysis of the TGFB1 and CXCL5 expression in LCN2 silencing DLD-1 cells. **(F)** WB and RT-qPCR analysis of the TGFB1 and CXCL5 expression in LCN2 overexpressing HCT-8 cells. Data are presented as the mean ± s.d. *p < 0.05, **p < 0.01, ***p < 0.001.

To further investigate the underlying molecular mechanisms of LCN2 in inhibiting CRC metastasis, RNA sequencing (RNA seq) was performed in DLD-1-LV-shLCN2 and DLD-1-LV-shNC cells. There were 561 differentially expressed genes from DLD-1-LV-shLCN2 and DLD-1-LV-shNC cells, among which 355 genes were upregulated and 206 genes were downregulated (Fold Change > 2, *p* < 0.05). Gene ontology (GO) analysis indicated that knockdown of LCN2 could positively regulate cell migration (**Figure 3A**). Kyoto Encyclopedia of Genes and Genomes (KEGG) pathway enrichment analysis showed that the knockdown of LCN2 was related to multiple pathways, including estrogen signaling pathway, MAPK signaling pathway, TNF signaling pathway, and ErbB signaling pathway (**Figure 3B**).

From a biological perspective, the transcriptome is the intermediate state of gene expression, whereas proteins are the direct functional performers of the organism; therefore, studying protein expression levels is indispensable (29). Next, we performed proteome analysis using DLD-1-LV-shLCN2 and DLD-1-LV-shNC cells, and 173 upregulated and 117 downregulated proteins were identified (Fold Change > 2, *p* < 0.05) (**Figure 3C**). We downloaded STAR-counts data and corresponding clinical information for CRC from the TCGA database (https://portal.gdc.cancer.gov). We then extracted data in TPM format and performed normalization using the log2(TPM+1) transformation. After retaining samples that included both RNAseq data and clinical information, we ultimately selected 620 samples for further analysis. We collected the genes included in the corresponding pathways and then analyzed them using the GSVA package in R software, choosing the parameter method=‘ssgsea’ for single-sample gene set enrichment analysis (ssGSEA). Finally, we studied the correlation between gene expression and pathway scores through Spearman correlation analysis. Statistical analysis was conducted using R software, version v4.0.3. Results were considered statistically significant when the p-value was less than 0.05. (**Figure 3D**). Among these candidate targets, TGFB1 and CXCL5 were significantly increased by knockdown of LCN2. RT-qPCR and WB assays further confirmed that knockdown of LCN2 significantly upregulated the mRNA and protein expression levels of TGFB1 and CXCL5, whereas the ectopic expression of LCN2 markedly reduced the mRNA and protein expression levels of TGFB1 and CXCL5 (**Figures 3E, F**). Based on these results, it can be inferred that the effects of LCN2 on CRC metastasis may be mediated by TGFB1 and CXCL5.

### 3.4 TGFB1 and CXCL5 act as downstream effectors of LCN2 to facilitate CRC metastasis

**Figure 4:**
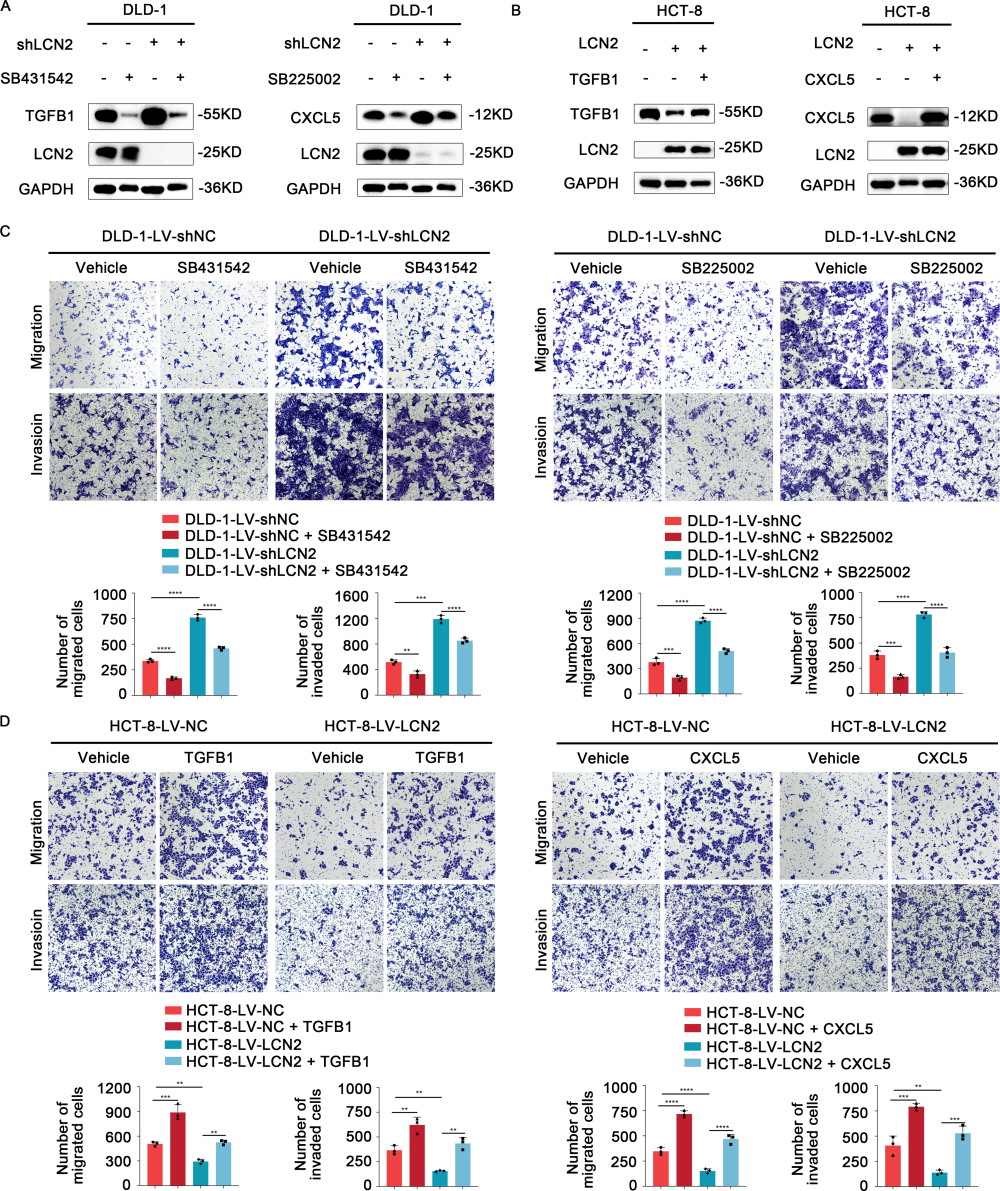
TGFB1 and CXCL5 act as downstream effectors of LCN2 to facilitate CRC metastasis. **(A)** WB analysis showed that TGF-βR1 inhibitor SB431542 or CXCR2 antagonist SB225002 could significantly weaken the expression level of intracellular TGFB1 or CXCL5, respectively. **(B)** WB analysis showed that recombinant human TGFB1 or CXCL5 protein could significantly enhance the expression level of intracellular TGFB1 or CXCL5, respectively. **(C)** Representative images from transwell assays showed the migratory and invasive capabilities of DLD-1-LV-shNC/shLCN2 cells after treatment with TGF-βR1 inhibitor SB431542 and CXCR2 antagonist SB225002. **(D)** Representative images from transwell assays showed the migratory and invasive capabilities of HCT-8-LV-NC/LCN2 cells after treatment with recombinant human TGFB1 or CXCL5 protein. Data are presented as the mean ± s.d. **p < 0.01, ***p < 0.001, ****p < 0.0001.

SB431542, a specific and potent inhibitor of TGFβ Receptor type I (TGF-βR1), could effectively block the TGFB1-mediated canonical pathway (30, 31). SB225002, as a CXCR2 selective antagonist, was used to block CXCL5 signaling (32). To further verify the role of TGFB1 and CXCL5 in LCN2-related CRC migration and invasion, DLD-1-LV-shLCN2 and DLD-1-LV-shNC cells were administrated with TGF-βR1 inhibitor (SB431542) and CXCR2 antagonist (SB225002), respectively. According to the results of WB, SB431542 could significantly decrease the intracellular expression of TGFB1, and SB225002 could significantly decrease the intracellular expression of CXCL5 (**Figure 4A** **).** Transwell assays demonstrated that treatment with either SB431542 or SB225002 significantly reduced the migratory and invasive capacities of DLD-1-LV-shNC cells and impaired the enhanced migratory and invasive capacity of DLD-1-LV-shLCN2 cells (**Figure 4C**). Subsequently, HCT-8-LV-NC and HCT-8-LV-LCN2 cells were individually treated with TGFB1 protein and CXCL5 protein, which could increase the level of intracellular TGFB1 or CXCL5 (**Figure 4B**). Transwell assays illustrated that TGFB1 or CXCL5 treatment potentiated the migratory and invasive capacities of HCT-8-LV-NC cells and mitigated the reduced migratory and invasive capabilities of HCT-8-LV-LCN2 cells (**Figure 4D**). Overall, these results indicated that TGFB1 and CXCL5 acted as downstream effectors of LCN2 to regulate CRC metastasis.

### 3.5 LCN2 inhibits CRC metastasis via the TGFB1/CXCL5 axis and the combination of TGF-βR1 inhibitor and CXCR2 antagonist mitigates LCN2-related CRC metastasis

**Figure 5:**
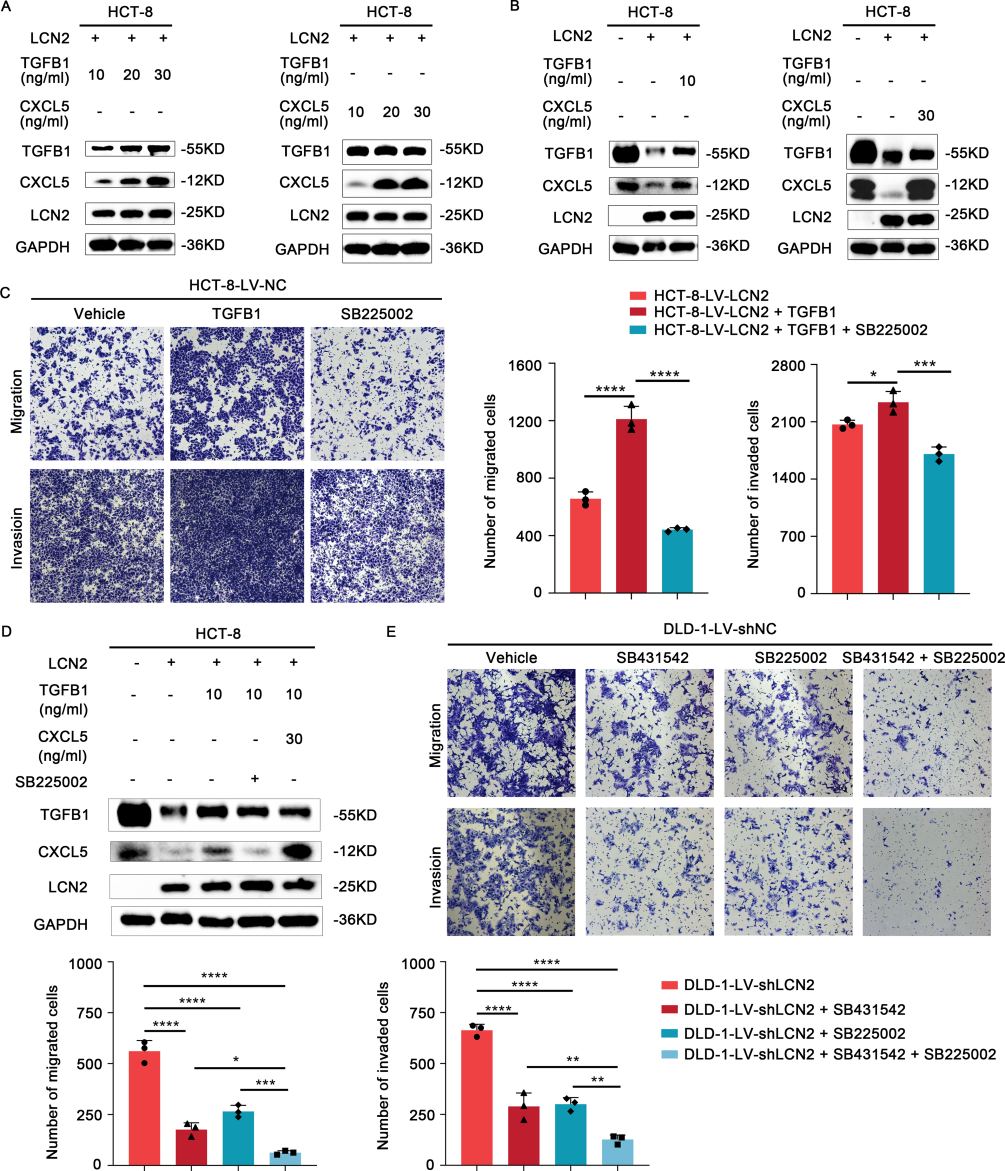
LCN2 inhibits CRC metastasis via the TGFB1/CXCL5 axis. **(A, B)** Western blot analysis demonstrates a marked increase in the protein levels of CXCL5 with varying concentrations of recombinant human TGFB1 protein. Conversely, the protein levels of TGFB1 remain largely unchanged across varying concentrations of recombinant human CXCL5 protein. **(C)** Representative images from transwell assays illustrate the migratory and invasive capabilities of HCT-8-LV-LCN2 cells after treatment with recombinant human TGFB1 alone or in combination with SB225002 (CXCR2 antagonist). **(D)** WB analysis showed that TGFB1 is a potential positive regulator upstream of CXCL5. **(E)** Representative images from transwell assays showed the migratory and invasive capabilities of DLD-1-LV-shLCN2 cells after treatment with TGF-βR1 inhibitor SB431542, CXCR2 antagonist SB225002 or the combination of both agents. Data are presented as the mean ± s.d. *p < 0.05, **p < 0.01, ***p < 0.001, ****p < 0.0001.

**Figure 6:**
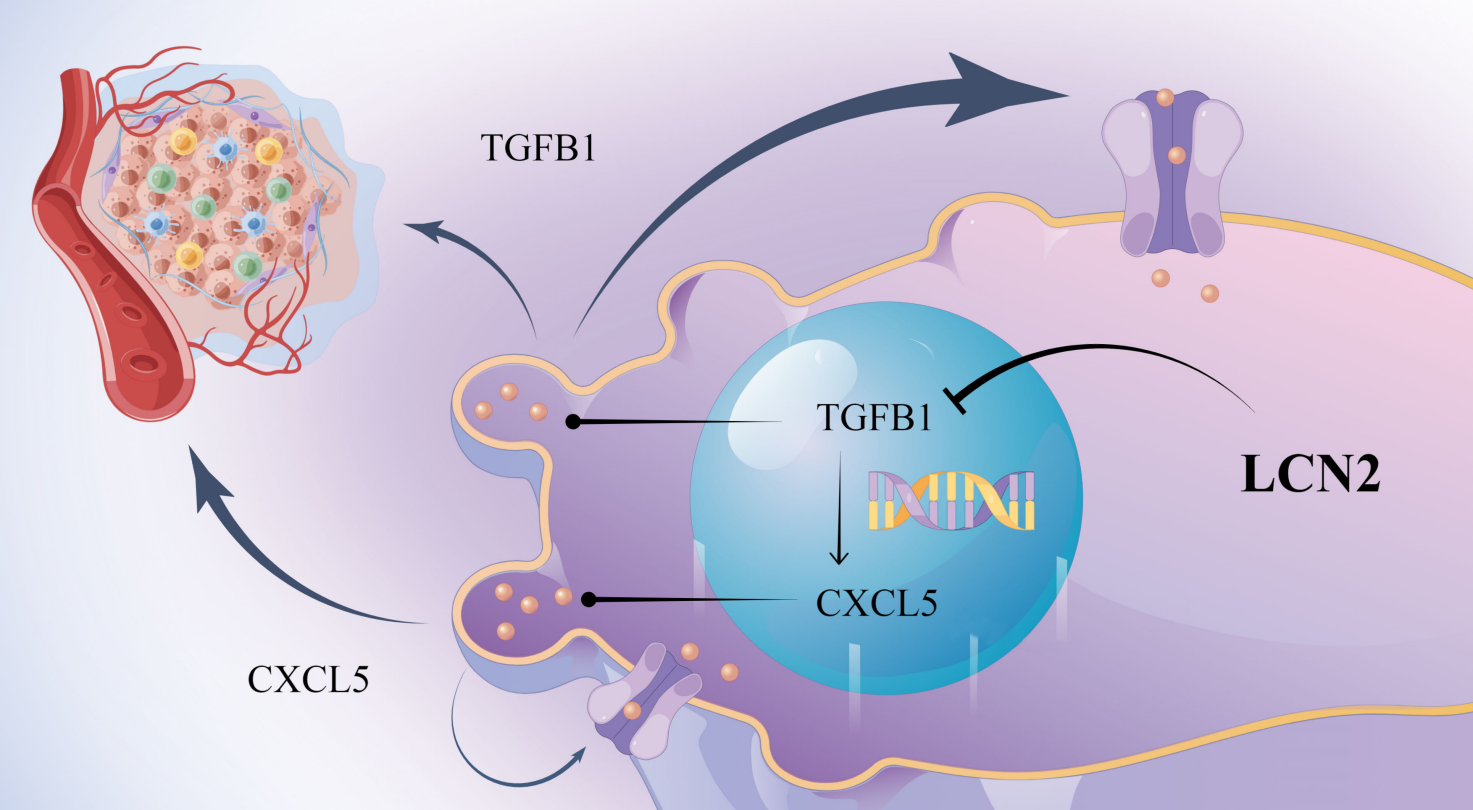
Schematic representation of LCN2/TGFB1/CXCL5 signaling axis created using Figdraw.

To determine the optimal concentration of TGFB1 and CXCL5, HCT-8-LV-NC and HCT-8-LV-LCN2 cells were treated with different concentrations. Interestingly, we observed that the expression of CXCL5 significantly increased with increasing concentration of TGFB1. However, the expression of TGFB1 was not altered with increasing concentration of CXCL5 (**Figures 5A, B, D**). Based on this observation, we speculated that CXCL5 might be a downstream effector of TGFB1. To further substantiate this speculation, we exposed HCT-8-LV-LCN2 cells, which had been treated with TGFB1 with 10ng/ml for 24h, to SB225002 (100μg/ml, 24h). Transwell assays demonstrated that TGFB1 enhanced the migratory and invasive capabilities of HCT-8-LV-LCN2 cells, whereas SB225002 significantly attenuated the migratory and invasive properties promoted by TGFB1 (**Figure 5C**). These data suggested that CXCL5 acts as a crucial functional downstream effector of TGFB1 in CRC metastasis. More importantly, a novel critical signaling axis LCN2/TGFB1/CXCL5 was identified to be involved in the regulation of CRC metastasis (**Figure 6**).

In light of our above findings, we hypothesized that the combination of SB431542 and SB225002 might have synergistic effects on metastasis treatment in CRC. DLD-1-LV-shLCN2 cells were administrated with SB431542 or SB225002, or both agents. Transwell assays suggested that either SB431542 or SB225002 treatment moderately decreased the migratory and invasive capabilities of DLD-1-LV-shLCN2 cells, whereas the combination treatment of the two agents significantly reduced the migratory and invasive capabilities of DLD-1-LV-shLCN2 cells (**Figure 5E**). Taken together, combined administration of TGFB1 inhibitor SB431542 and CXCR2 antagonist SB225002 remarkably suppressed LCN2-related CRC metastasis.

## 4 Discussion

In recent years, continuous advancements in surgical techniques and the widespread adoption of high-quality screening programs have translated into improved survival rates of patients with CRC (33, 34). Nevertheless, distant metastasis remains the primary contributor leading to poor clinical prognosis, with 5-year survival rates decreasing from 91% for early-stage disease to 14% for advanced metastatic disease (3). Thus, elucidating the molecular mechanisms of metastasis is essential for the development of targeted therapeutic strategies.

In this study, analysis of data from GEO and GEPIA databases revealed that the expression level of LCN2 in CRC tissues was upregulated and subsequently validated via RT-qPCR, WB, and immunohistochemical assays. Furthermore, we assessed the relationship between clinical follow-up data and immunohistochemical score, which demonstrated that LCN2 was significantly correlated with AJCC stage, N classification, and OS. More importantly, multivariate analysis showed that the high expression of LCN2 was an independent protective factor for OS. Next, LCN2 overexpression and knockdown cell lines were constructed via lentiviral vectors to perform gain- and loss-of-function experiments, demonstrating that LCN2 could serve as a tumor suppressor gene in CRC metastasis. By analyzing transcriptomic and proteomic results and validation by RT-qPCR, WB, and rescue experiments, TGFB1 and CXCL5 were identified as downstream targets of LCN2. Interestingly, we found that CXCL5 is a downstream target of TGFB1, forming the LCN2/TGFB1/CXCL5 axis, which suppresses CRC metastasis by inhibiting cell migration and invasion. Considering that TGFB1 and CXCL5 are promising therapeutic targets, we hypothesized that combining these two strategies may yield synergistic anticancer effects. Indeed, our results demonstrated limited efficacy when SB431542 or SB225002 was used in isolation, whereas their combined application exhibited significant synergy in suppressing CRC metastasis. Therefore, the co-administration of TGFB1 inhibitors and CXCL5 inhibitors for the treatment of LCN2-induced CRC metastasis emerges as a promising therapeutic strategy.

Although previous studies on LCN2 have demonstrated conflicting roles as an oncogene or a tumor suppressor gene in multiple malignancies, such as gastric cancer (16, 35, 36), breast cancer (15, 37), ovarian cancer (38), prostate cancer (39), oral squamous cell carcinoma (17), hepatocellular carcinoma (18), cholangiocarcinoma (40) and colorectal cancer (41), research on the tumor metastasis is far from sufficient particularly regarding the underlying mechanisms. In this study, we uncovered a novel regulatory axis, LCN2/TGFB1/CXCL5, expanding our understanding of LCN2’s role in CRC. In addition, numerous studies have revealed that the expression of CXCL5 is widely upregulated in malignant tumors, which could be responsible for the malignant phenotypes, including lymphatic metastasis (42), tumor angiogenesis (43), proliferation (44), migration and invasion (25–27, 44, 45). However, the mechanisms of its overexpression in malignant tumors have rarely been reported. Herein, we found that CXCL5 overexpression in CRC was caused by the downregulation of LCN2. Furthermore, there is increasing evidence suggesting that LCN2 is responsible for malignant phenotypes via activating various signaling pathways (18, 41, 46). In recent years, research on the distinct role of the TGF-β signaling pathway in cancer progression has gained significant momentum, including EMT and apoptosis (47), chemoresistance (48), angiogenesis (49), and metastasis (48, 50). Our findings suggest that LCN2 may suppress CXCL5 expression by inhibiting the TGF-β signaling pathway, serving as a bridge between these key elements. However, another study has indicated that LCN2 is a downstream target of TGFB1, leading to the downregulation of Twist1 (46). It is highly conceivable that there is a negative feedback loop between LCN2 and TGFB1. The mechanisms behind this interesting phenomenon are worth further exploring.

In recent years, the functions of LCN2 in the metastatic phenotype of malignant tumors have attracted a significant amount of interest (15–17, 35, 41). It is widely thought that LCN2 is mainly involved in regulating tumor metastasis by promoting or suppressing the progression of EMT and MET. The metastasis of cancers can be attributed to their complex ecosystem of cells with heterogeneous functional states, which is known as the TME (51). The cellular composition and functional status of TME may vary considerably depending on the organ in which the tumor arises, the intrinsic characteristics of the cancer cells, the stage of the tumor, and patient characteristics (51). Combining the multiple physiological and pathophysiological functions of LCN2, we speculated that LCN2 might be involved in metastatic progression by regulating the TME. First, the presence of iron is essential for the growth of almost all organisms and its homeostasis is tightly associated with inflammation and cancer (8, 52). LCN2 specifically binds to siderophores (53), altering intracellular iron levels (54), which plays a critical regulatory role in tumor proliferation and metastasis (52, 55) via altering TME-cells survival status. Besides, LCN2 is closely related to ferroptosis (56, 57), which is a phenotype of cell death (58). The secreted LCN2 plays a crucial role in inducing the ferroptosis and wasting of adipose and muscle tissues in lung cancer cachexia (56). It has been reported that upregulation of LCN2 could deplete iron and weaken sensitivity to ferroptosis inducers in liver cancer cells (57). The above studies overlap in their assertion that LCN2 could regulate the ferroptosis process of both tumor cells and TME cells, ultimately playing a synergistic role in promoting CRC metastasis. Furthermore, tumor-associated macrophages (TAM) are the primary source of iron for the tumor (59). Besides, the upregulation of LCN2 in TAM contributes to the enhancement of iron release from TAM to the TME, enhancing tumor growth (60). The study by Chaudhary et al (61), indicated that the cell death induced by 5-fluorouracil (5FU) partially depends on the ferroptosis pathway, while LCN2 protects tumor cells through the aforementioned mechanisms, resulting in therapeutic resistance. LCN2 suppresses ferroptosis via dual mechanisms—iron metabolism regulation and antioxidant gene expression—thereby driving chemoresistance and tumor progression in colorectal cancer. The monoclonal antibody targeting LCN2 provides a novel strategy to overcome therapeutic resistance and demonstrates significant potential for clinical translation. Taken together, these findings suggest that LCN2 may regulate CRC metastasis by exerting bidirectional effects on both tumor cells and TME cells, and the LCN2/TGFB1/CXCL5 axis discovered in this study represents just a fraction of its complex involvement. Further in-depth investigations are warranted to unravel these intricate interactions in the future.

Nonetheless, our study possesses limitations that require further attention. Firstly, although we established that LCN2 is overexpressed in CRC, the regulatory mechanisms involving the upstream molecule PUS7 remain unknown. The specific molecular mechanisms by which LCN2 regulates TGFB1 and CXCL5 were not fully elucidated. Secondly, the relatively small number of patients included in the CRC tissue microarray may introduce limitations to our outcomes. The absence of *in vivo* experiments investigating TGF-βR1 inhibitor SB431542 and CXCR2 antagonist SB225002-based therapeutic approaches significantly compromises the clinical relevance of the LCN2-TGFB1-CXCL5 regulatory axis. Thirdly, *in vitro*-cultured CRC cells may not fully recapitulate the TME that operates *in vivo*. Fourthly, the specific molecular mechanisms by which LCN2 regulates TGFB1 were not fully elucidated. Additionally, the regulatory mechanisms of the TGF-β pathway on CXCL5, a downstream target molecule, remain unclear and require further in-depth investigation. Finally, our study has focused solely on one phenotype of LCN2 in CRC—metastasis, while other tumor-progression-influencing phenotypes such as proliferation and apoptosis still require in-depth investigation to explore LCN2’s broader potential and clinical significance.

## 5 Conclusions

In the present study, we confirmed that LCN2 exhibits high expression levels in CRC tissues, and LCN2 expression independently predicted a more favorable outcome for CRC patients. Upregulation of LCN2 effectively suppressed CRC cell metastasis both *in vitro* and *in vivo.* TGFB1 and CXCL5 were identified as downstream target genes of LCN2, and rescue experiments verified the necessity of TGFB1 and CXCL5 in LCN2-mediated CRC cell migration and invasion. Combined treatment of SB431542 and SB225002 dramatically decreased LCN2-related CRC metastasis. Thus, we elucidated the molecular mechanism of LCN2 inhibiting the migration and invasion of CRC cells and clarified that targeting the TGFB1/CXCL5 axis may be a novel approach for LCN2-related CRC therapy.

## Data Availability

The datasets presented in this study can be found in the GEO repositories. The names of the repository/repositories and accession number(s) can be found below: https://www.ncbi.nlm.nih.gov/geo/, GSE294201.
