# Supplementary figures and images for: TGFB1/CXCL5 axis regulation by LCN2 overexpression: a promising strategy to inhibit colorectal cancer metastasis and enhance prognosis
Source: Front Immunol. 2025 Apr 17;16:1548635. doi: 10.3389/fimmu.2025.1548635 (PMC12043584; doi:10.3389/fimmu.2025.1548635)

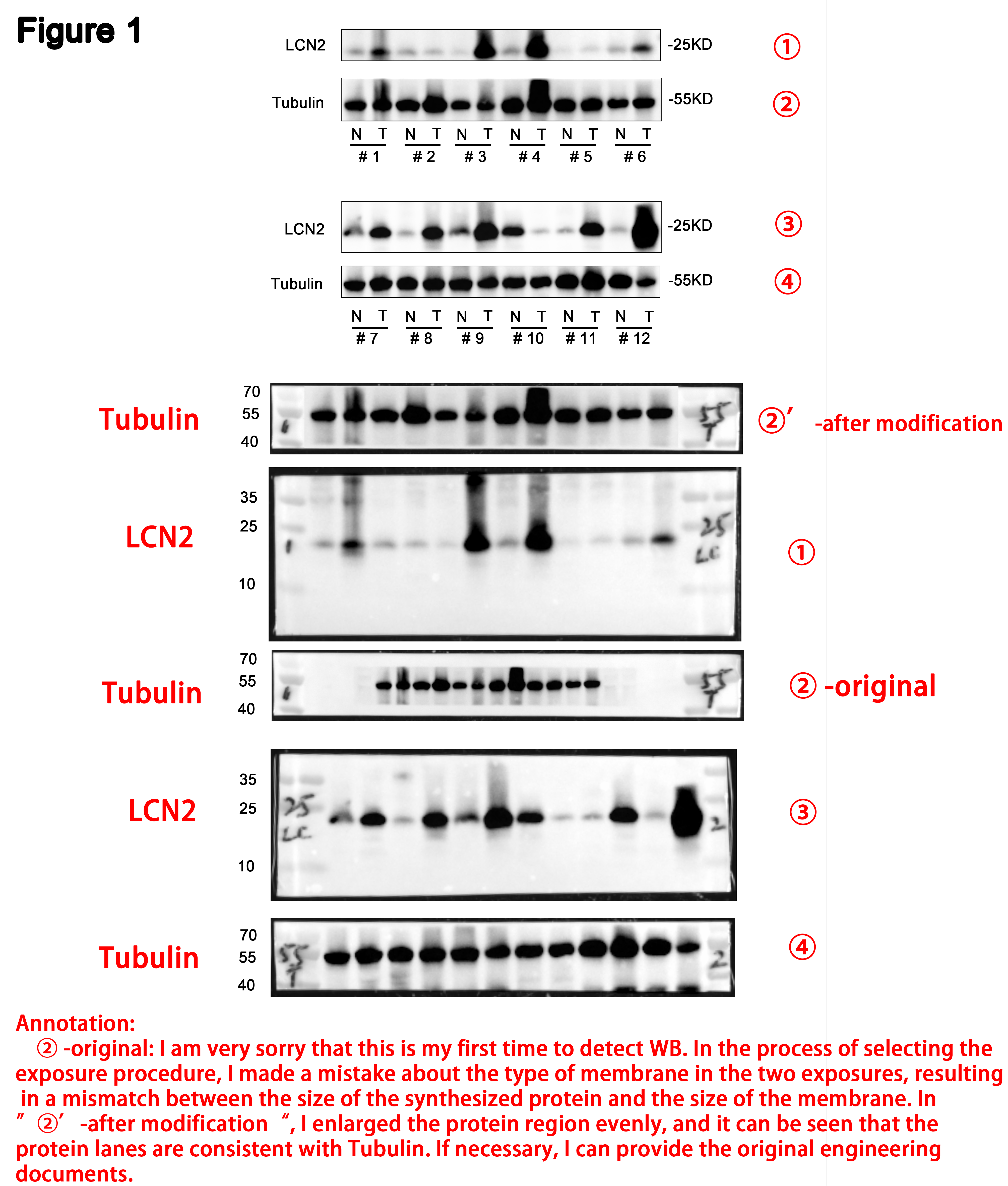

Supplement: Supplementary file 4 [file Image1.tif]

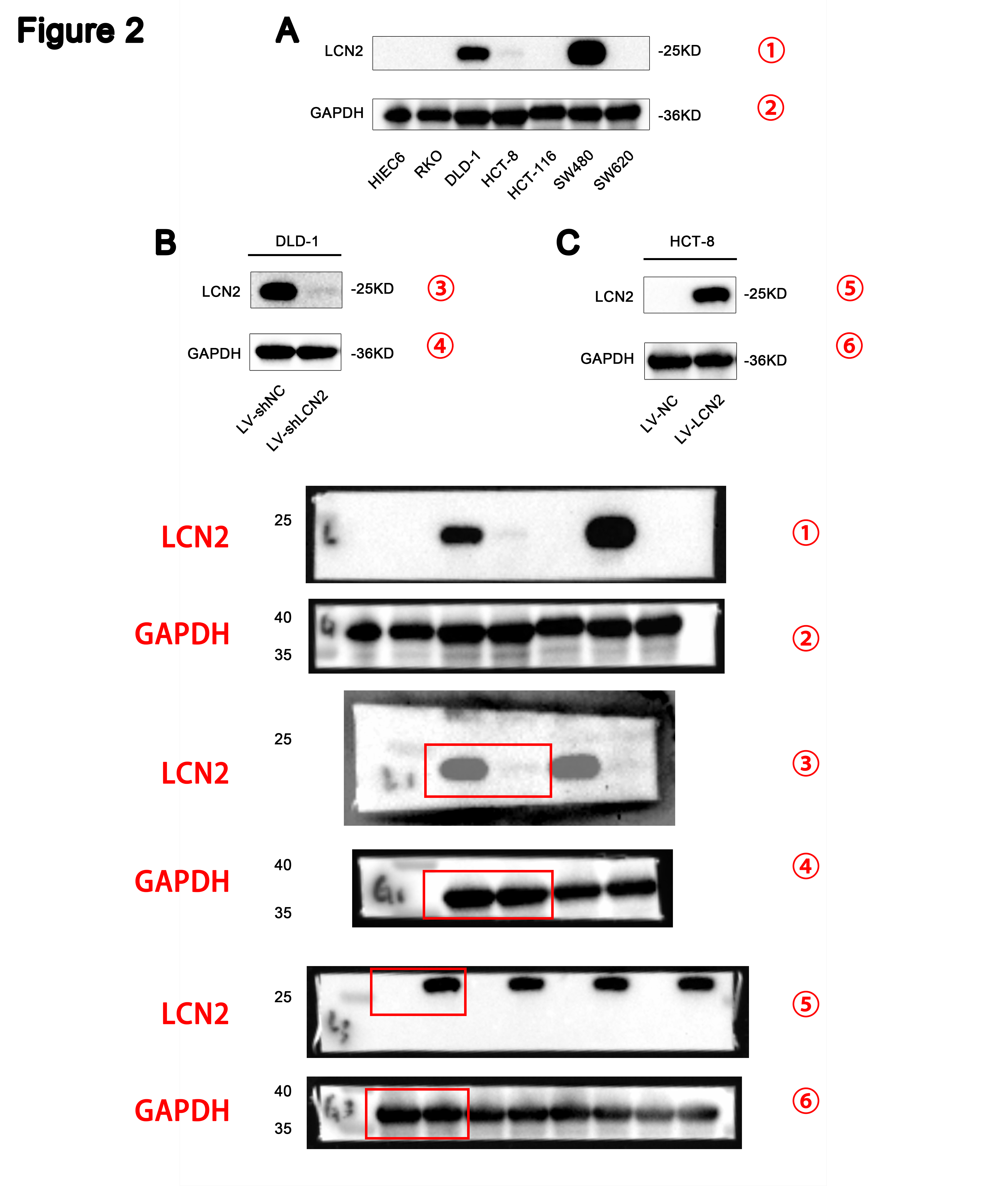

Supplement: Supplementary file 5 [file Image2.tif]

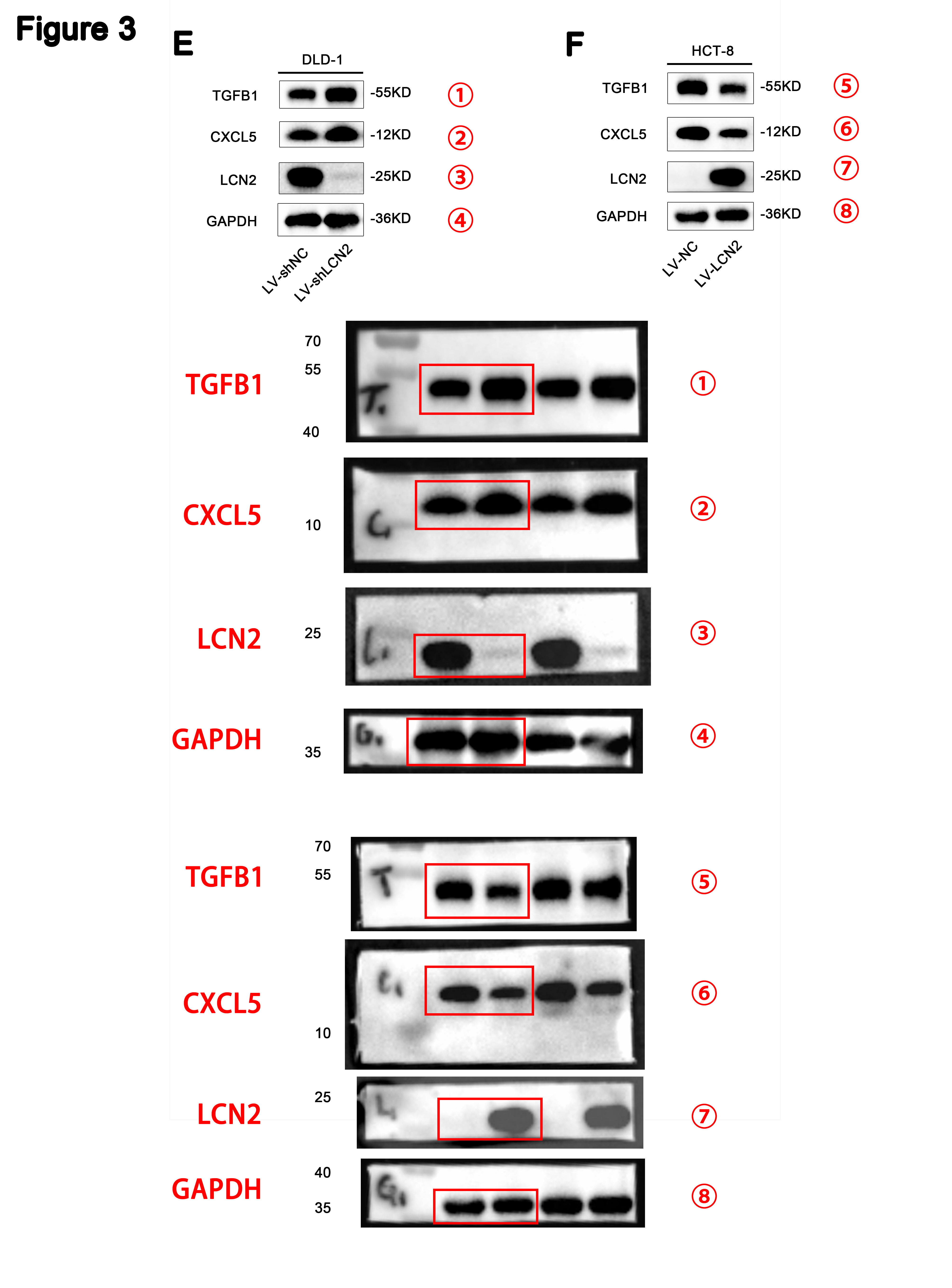

Supplement: Supplementary file 6 [file Image3.tif]

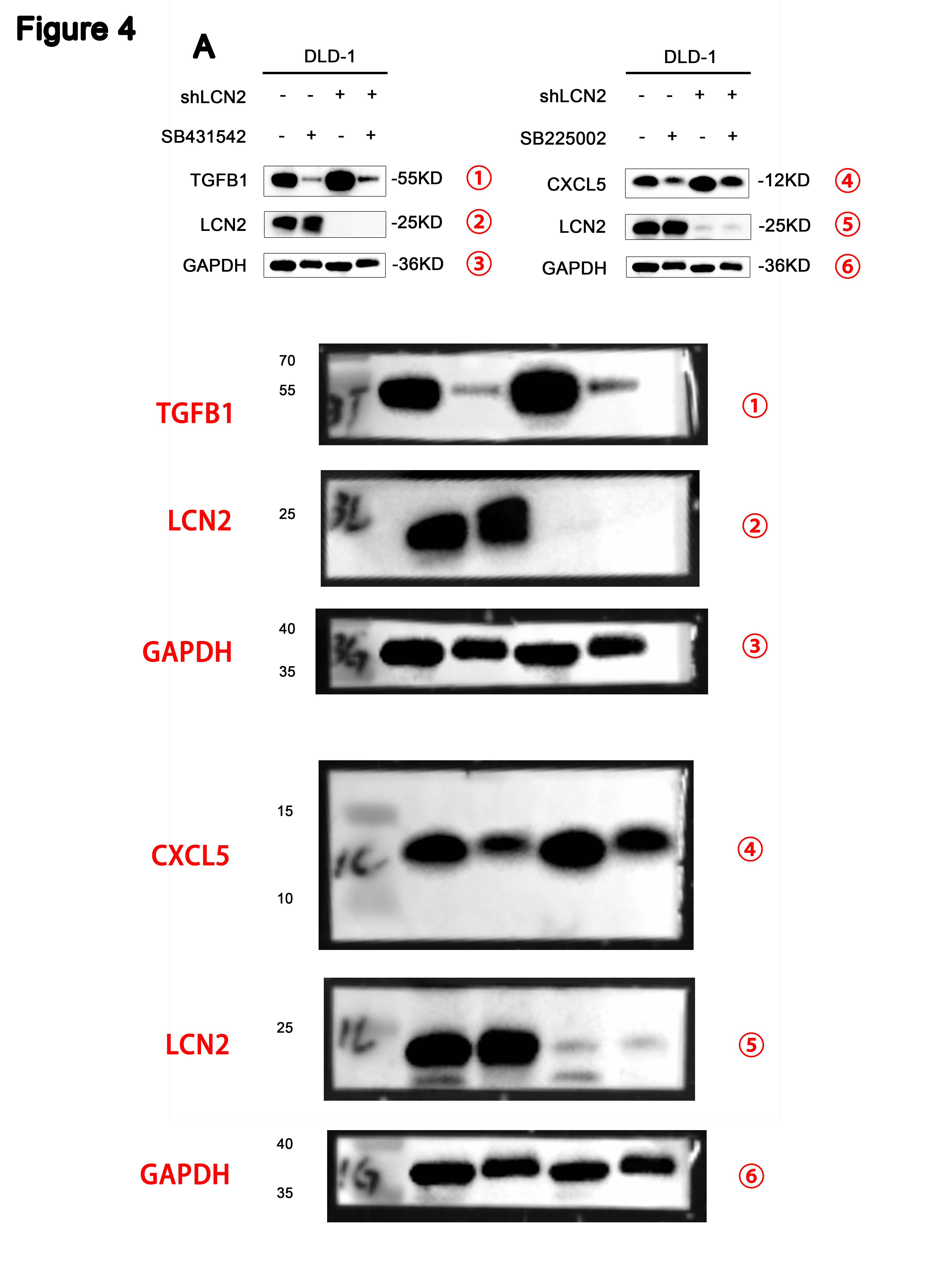

Supplement: Supplementary file 7 [file Image4.tif]

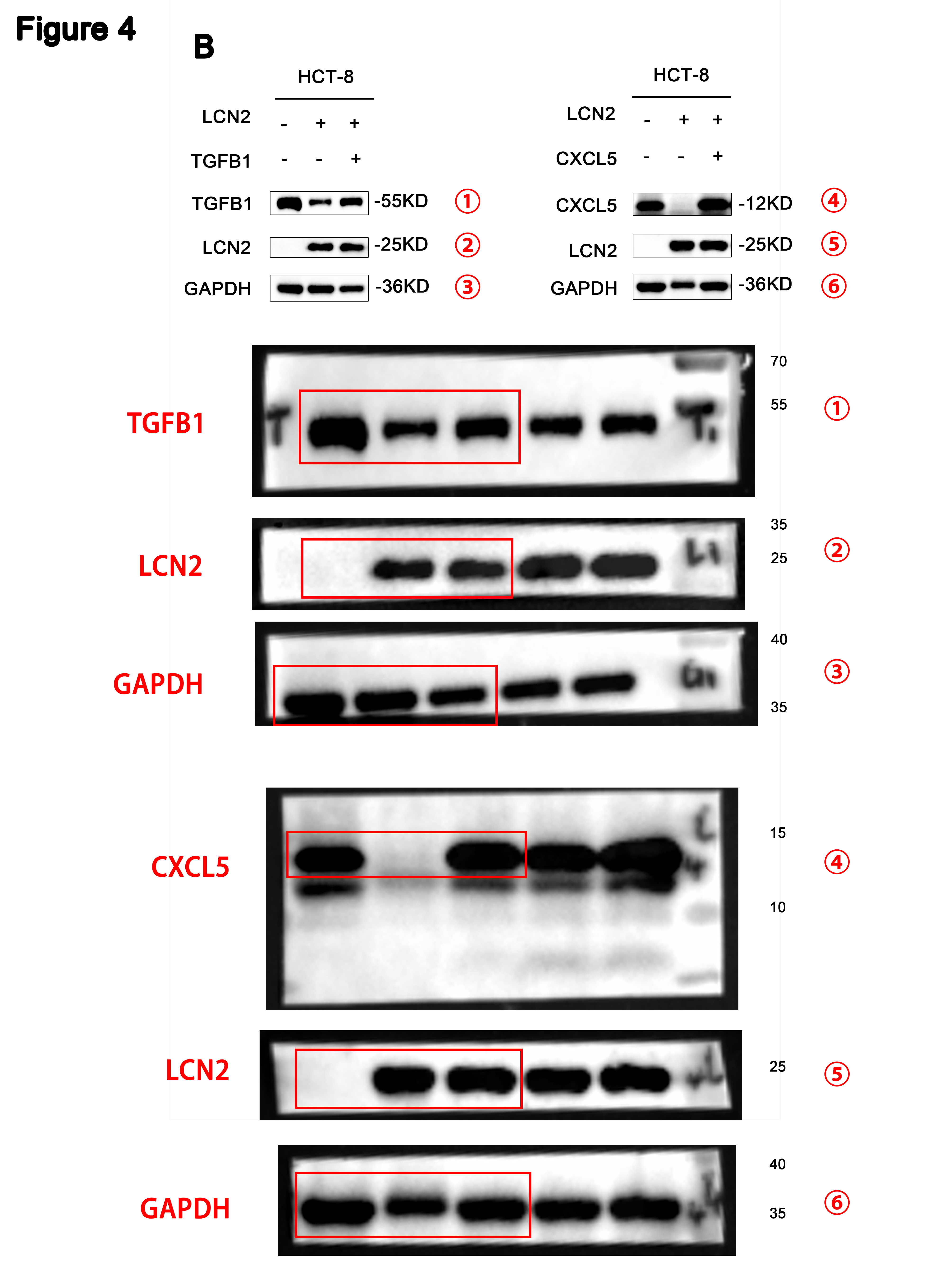

Supplement: Supplementary file 8 [file Image5.tif]

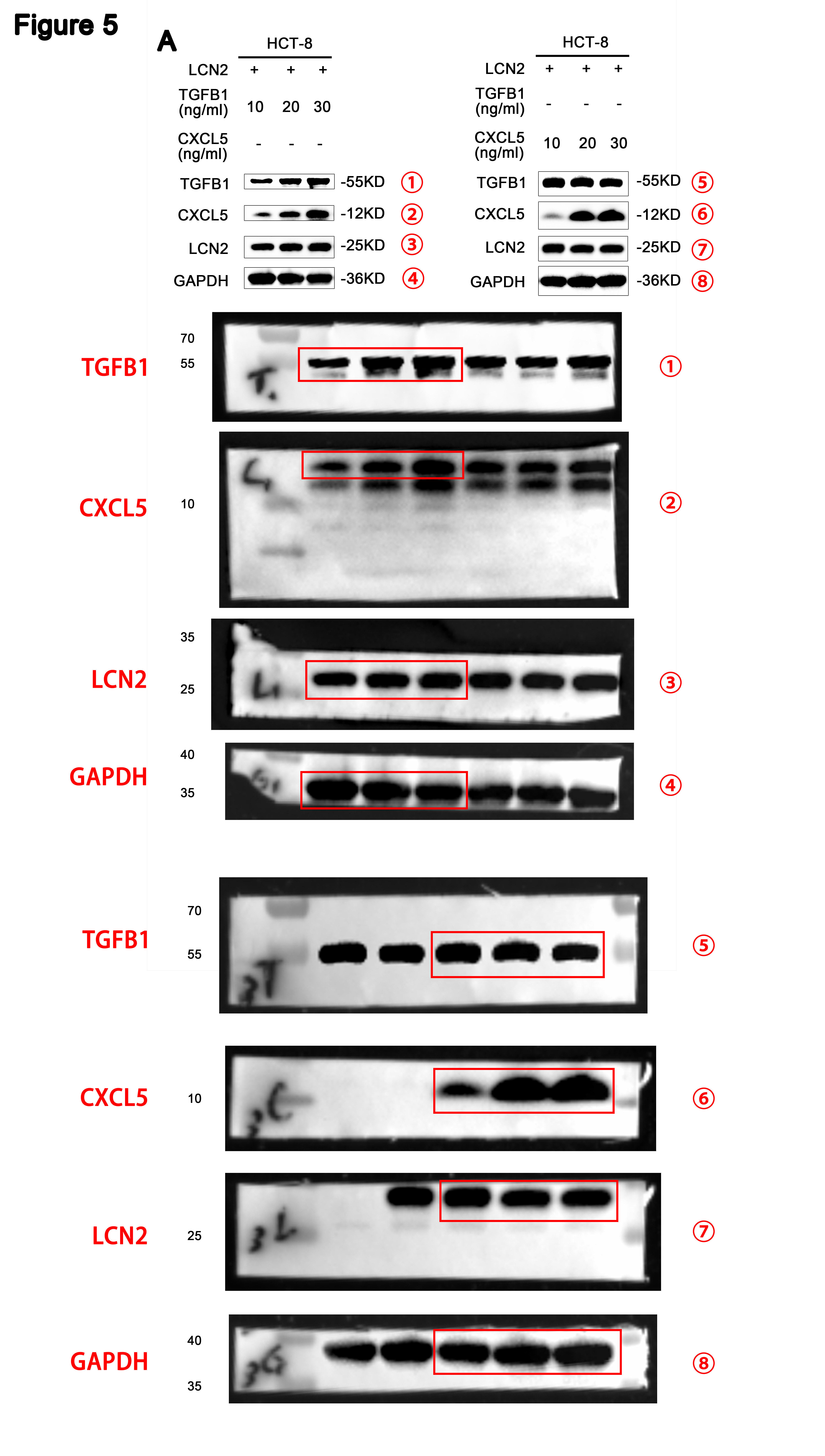

Supplement: Supplementary file 9 [file Image6.tif]

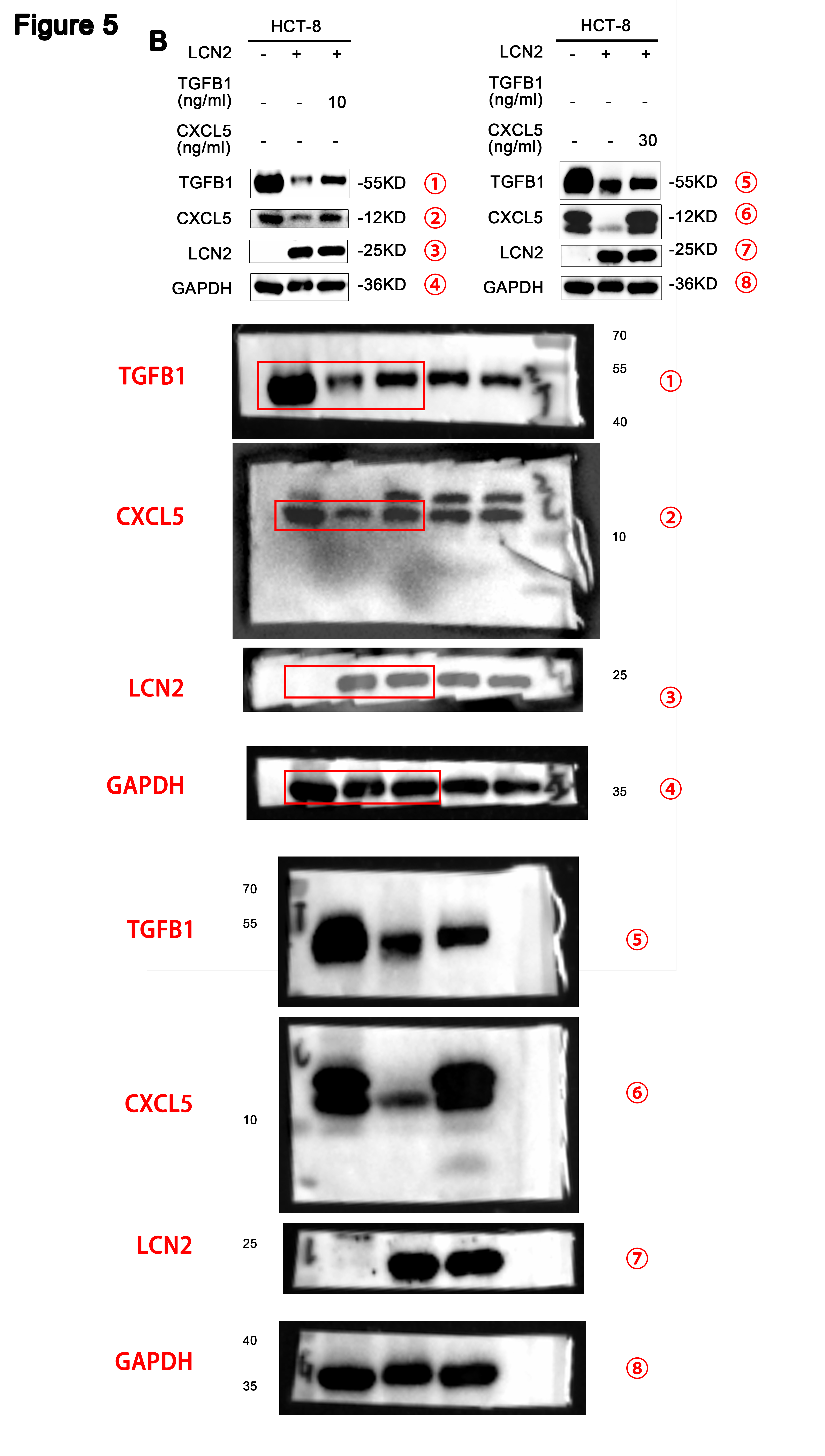

Supplement: Supplementary file 10 [file Image7.tif]

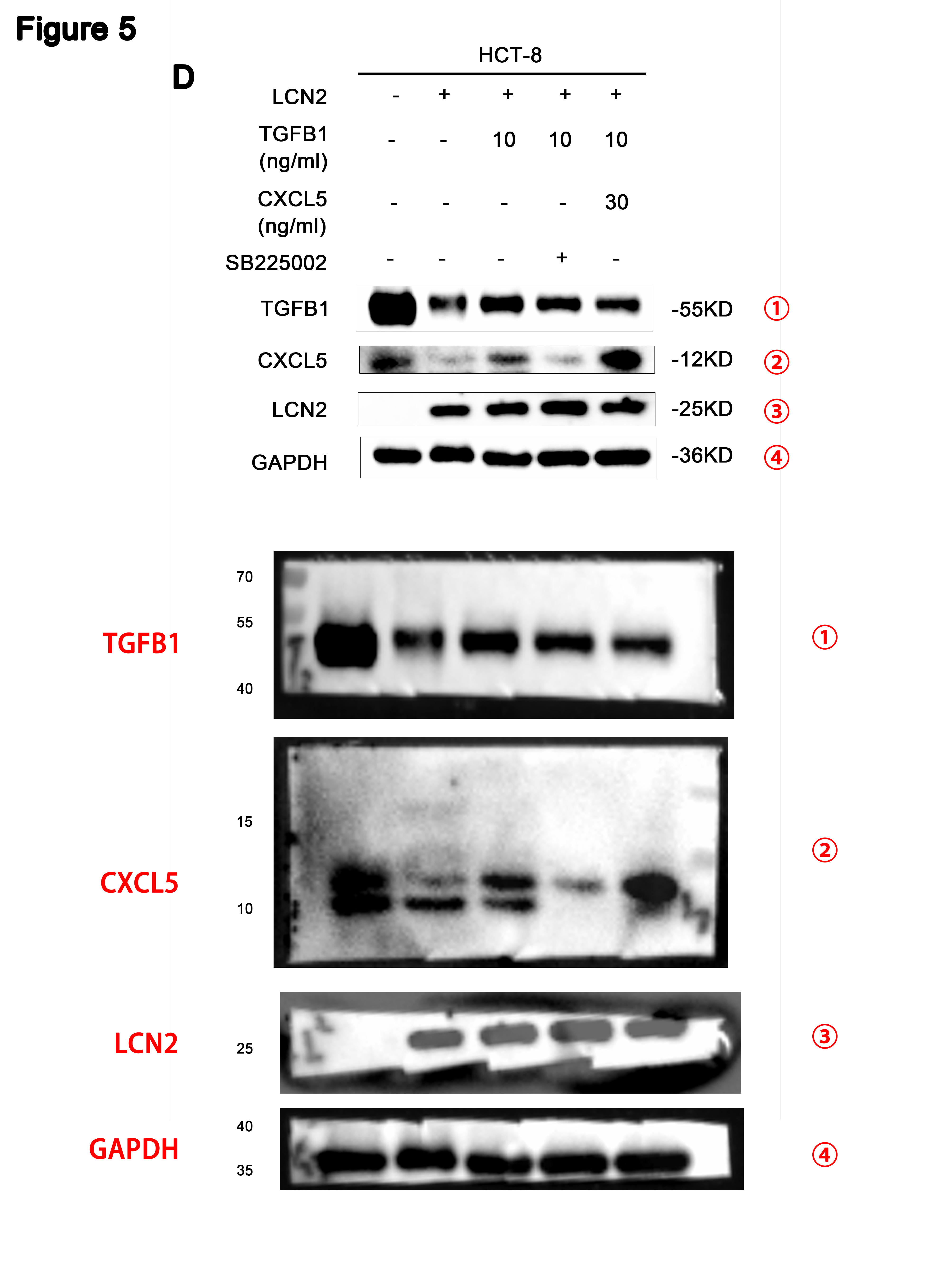

Supplement: Supplementary file 11 [file Image8.tif]
